# Perspectives for glycaemic control in type 2 diabetes in Kinshasa, Democratic Republic of the Congo

**DOI:** 10.1093/heapro/daad128

**Published:** 2023-10-10

**Authors:** Jean-Pierre Fina Lubaki, Joel Msafiri Francis, Olufemi Babatunde Omole

**Affiliations:** Department of Family Medicine and Primary Care, University of the Witwatersrand, Phillip V Tobias Health Sciences Building, 29 Princess of Wales, Parktown, Johannesburg 2193, South Africa; Department of Family Medicine and Primary Care,Protestant University of Congo, Croisement des Avenues Libération et Triomphale, Commune de Lingwala, Kinshasa, Democratic Republic of the Congo; Department of Family Medicine and Primary Care,University of the Witwatersrand, Phillip V Tobias Health Sciences Building, 29 Princess of Wales, Parktown, Johannesburg 2193, South Africa; Department of Family Medicine and Primary Care,University of the Witwatersrand, Phillip V Tobias Health Sciences Building, 29 Princess of Wales, Parktown, Johannesburg 2193, South Africa

**Keywords:** glycaemic control, perspectives, type 2 diabetes, qualitative study, sub-Saharan Africa

## Abstract

Glycaemic control is a significant problem in the Democratic Republic of the Congo (DRC), the perspectives associated with glycaemic control are not fully known as previous studies rarely explored patients’ perspectives and lived experiences. This qualitative study described the perspectives regarding glycaemic control among persons with type 2 diabetes in Kinshasa, DRC. A total of 23 participants were purposively selected in seven health centres in Kinshasa. In-depth interviews were used for data collection. The study used a phenomenology approach, and deductive, constructionist and thematic analysis. Data analysis was performed using the MAXQDA 2022. Five themes were identified as perspectives for glycaemic control in Kinshasa: financial constraints, limited social and relational support, difficulties with lifestyle changes, beliefs and practices about diabetes and ability to adapt for caring for the illness. Themes were integrated using social cognitive theory. Participants expressed that they were unable to achieve better glycaemic control due to financial constraints, limited social and relational support and difficulty in changing their lifestyle. Their beliefs and practices about diabetes also constituted a barrier. Our results showed that lack of adequate funding is a major determinant of glycaemic control and therefore it is crucial to integrate a consistent and reliable funding system for care of people living with diabetes. Persons with diabetes must be empowered to successfully adapt to the requirements of diabetes care. In this process, support for people living with type 2 diabetes is also essential and should involve their families as well as healthcare providers.

**Contribution to Health Promotion**

- Glycaemia control is a multifactorial problem requiring greater investment in health by society and equity in the repartition of resources.
- Better preparation and fundamental changes in the healthcare system are essential to meet the challenges of chronic diseases.
- People living with diabetes must be encouraged and equipped with the necessary skills for effective self-care.

## BACKGROUND

About 537 million adults (20–79 years) are living with diabetes worldwide, and this number is expected to rise to 643 million by 2030 (International Diabetes Federation., 2022a). Of these, over 3 in 4 adults with diabetes will live in low- and middle-income countries. In Africa, diabetes was responsible for 416 000 deaths in 2021 and 54% of people living with diabetes are undiagnosed (International Diabetes Federation., 2022a).

The World Health Organization (WHO) targets adopted by the 75th World Health Assembly in 2022 stated that 80% of people diagnosed must have good control of glycaemia (WHO, 2022), but worldwide, glycaemic control remains a challenge for all the stakeholders engaged in the care of diabetes (Giugliano *et al.*, 2018).

In sub-Saharan Africa, studies have shown high rates of poor glycaemic control among patients with type 2 diabetes in several settings. The DiabCare study conducted in six sub-Saharan countries—Cameroon, Ghana, Kenya, Nigeria, Senegal and Tanzania—found that only 29% of the participants reached a level of glycosylated haemoglobin below 6.5% (Sobngwi *et al.*, 2012). Asystematic review in Ethiopia found that only 33.2% (95% CI: 21.8–44.6) of the participants had good glycaemic control (Gebreyohannes *et al.*, 2019). A metaanalysis of glycaemic control in sixteen sub-Saharan African countries showed that only 30.3% (95% CI: 27.6–32.9) of the participants had good glycaemic control (Fina Lubaki *et al.*, 2022A). Therefore, persons with diabetes are exposed to complications and increased costs of diabetes care in a context characterized by high levels of poverty and resource constraints. In a recent systematic review, the factors associated with poor glycaemic control included age, gender, lower income, absence of health insurance, low level of education, place of residence, family history of diabetes, longer duration of diabetes, high pill burden, treatment regimen, side effects, use of statins or antihypertensive medications, alcohol consumption, smoking, presence of comorbidities/complications and poor management (Fina Lubaki *et al.*, 2022A). Very few studies have been conducted to assess the patients’ perspectives on glycaemic control leading to a gap in knowledge (Adeniyi *et al.*, 2015; Gathu *et al.*, 2018). In the Democratic Republic of Congo (DRC), diabetes affects an estimated 1 908 900 adults, representing a prevalence of 4.8% (International Diabetes Federation., 2022b). The country is a low-income country with one out of six individuals living in extreme poverty (World Bank, 2021). In Kinshasa, less than 10% of the population has employment and only a few are covered by health insurance (World Bank, 2021). In this setting, persons with diabetes and their families spend a large portion of their incomes to meet the costs of diabetes care, resulting in a large proportion not meeting glycaemic targets (Mottini *et al.*, 2003; Sagastume *et al.*, 2022). Failure to meet glycaemic targets on the other hand exposes persons with diabetes to complications of diabetes with the subsequent increased cost of care (Mottini *et al.*, 2003; Sagastume *et al.*, 2022). The challenges encountered by persons with type 2 diabetes in following their treatment deserve to be explored to improve treatment and, by extension, glycaemic control. However, most studies on glycaemic control in the DRC have excluded persons with diabetes’ perspectives in the complex process of diabetes care (Longo-Mbenza *et al.*, 2011; Sagastume *et al.*, 2022). The reasons for poor glycaemic control have not been fully explored in Kinshasa as the perspectives of persons with diabetes and their lived experiences of glycaemic control have rarely been assessed. This knowledge gap has clinical and public health policy implications in that interventions may be constructed outside of patients’ contexts and therefore less acceptable to patients and less scalable. Understanding the patients’ perspectives and experiences would be beneficial for the management of patients with diabetes (Zimmermann *et al.*, 2018). This study aims to describe the perspectives on glycaemic control among persons with type 2 diabetes. The results will contribute to building an intervention package for persons with type 2 diabetes in Kinshasa, DRC.

## METHODS

### Study design

This study engaged a qualitative approach to explore the perspectives of persons with type 2 diabetes mellitus regarding diabetes treatment in Kinshasa, DRC. A total of 23 participants were included in the study and one-on-one in-depth interviews were used to collect data. This study is part of a large project dealing with glycaemic control in Kinshasa (Fina Lubaki *et al.*, 2022B). A preprint of this study has been published (Fina Lubaki *et al.*, 2023). The study is reported according to the consolidated criteria for reporting qualitative research (COREQ) (Tong *et al.*, 2007).

### Study setting

This study was conducted in seven health centres in the city of Kinshasa, the capital of the DRC. Kinshasa has an estimated population of about 15 million and spreads over an area of 9965 km^2^ (World Bank, 2021). The seven health centres were Bondeko, 2eme Rue, Mokengeli, Saint Ambroise, Saint-Pierre, Etonga and Kikimi. These health centres are part of the Kinshasa Primary Healthcare Network reuniting hospitals and health centres mainly from the Catholic Church and Salvation Army (Kapongo *et al.*, 2015; Sagastume *et al.*, 2022). These health centres are distributed in the 24 health districts in Kinshasa and integrated diabetes care. Persons with type 2 diabetes did not have access to specialists in diabetes care; they were managed by generalists trained in diabetes care who visited them once monthly. Between visits, some persons with diabetes could return to the health centres for blood glucose monitoring and advice from the nurses. For this study, the seven health centres were purposively selected to ensure the diversity of patients in terms of socioeconomic, illness duration and experiential contexts. These were the facilities where patients who were likely to expressively share their perspectives have been identified and also provided a conducive environment for the interviews to be held.

### Study population, sample size and sampling

The study population comprised of persons with type 2 diabetes mellitus attending the seven selected healthcare facilities in Kinshasa, DRC. The inclusion criteria were age ≥18 years, being diagnosed with type 2 diabetes and being on treatment for at least 6 months. Pregnant women, those with difficulty communicating due to mental disability, and patients with diabetes emergencies were excluded.

Consecutive and purposefully selected information-rich persons with type 2 diabetes were recruited from the seven facilities to satisfy the need for diversity in sex, duration of diagnosis/illness, socioeconomic strata treatment categories and levels of glycaemic control (Vasileiou *et al.*, 2018). Health facilities’ teams who were well acquainted with the patients helped in their recruitment. The role of the health facilities teams was to point out persons with diabetes susceptible to effectively discuss their perspectives on glycaemic control. The members of health facilities teams were not involved in contacting the persons with diabetes for the study. Only the interviewer was in charge of presenting the study to the participants and inviting them to enter the study. Recruitment continued until data saturation. Data saturation was reached when no new element of the codes was identified (Hennink and Kaiser, 2022). After each interview, data was analysed to identify codes. Then in subsequent interviews, a search for new aspects, dimensions, or nuances of the codes was performed until novel findings were uncommon. In total, we conducted 23 interviews of persons living with type 2 diabetes. None of the patients approached refused to participate or were excluded.

### Data collection

The interviews were then conducted by the principal investigator in a quiet room where the confidentiality of the participant’s statements was guaranteed. Only the study staff was involved in the organization of the interviews. The interviews were one-on-one in-depth interviews. The interview was facilitated by exploring the clarity of the participants’ statements. The interviews were guided by an interview schedule (Supplementary File 1) and were conducted in French or Lingala depending on the preferences of the participants. All interviews were audio recorded and lasted at most 45 min. As the interviews were conducted during the COVID-19, there was a strict observation of the COVID-19 rules. The data collection took place from December 2021 to March 2022.

### Data management and analysis

The recorded interviews were transcribed verbatim and then translated into English by a language expert. The principal researcher who is fluent in French and Lingala verified the translation and transcription by re-listening to the audio while reading the transcripts. Data coding and thematic analysis were performed using the MAXQDA 2022 (Verbi Software, 2021). The analysis went through the six steps described by Braun and Clarke (Braun and Clarke, 2006)—familiarization with the data, generating initial codes, searching for themes, reviewing potential themes, defining and naming the themes and producing the report.

In MAXQDA, the researcher uploaded the transcripts of the interviews. When he identified a code during the readings, he created it in the system and wrote a memo to describe its meaning. After that, all the words, parts of sentences, sentences or groups of sentences related to this code were associated with this particular code. One may note that a sentence a word, part of a sentence, a sentence, a group of sentences could be associated with more than one code. Then, the different codes identified were grouped into sub-themes and finally into themes. The coding was performed independently by the principal researcher (J.P.F.) and one supervisor (O.B.O.) in selected interviews without any pre-conceptualized theoretical framework. The integration of the themes identified was performed using social cognitive theory (Thojampa and Sarnkhaowkhom, 2019; Sebastian *et al.*, 2021).

### Credibility, transferability, dependability and confirmability

The credibility of the study is a measure of the truth value of qualitative research, or whether the study’s findings are correct and accurate (Guba, 1981). In our study, it was enhanced by peer debriefing and member checking. Two experts, one in diabetology and another in sociology assisted in conceptualizing the findings of the study. The findings were submitted to five participants to seek their agreement with identified themes (member checking). Triangulation of findings between the principal investigator and the supervisors enhanced credibility. Transferability measures whether, or to what extent, the study’s results are applicable within other contexts or settings (Guba, 1981). It was ensured in this study by thick description or extensive description of the site, participants and the methods used during the study. Dependability is used to measure the consistency and reliability of the results (Guba, 1981). It is done by an exam of the methods used, the analysis and the interpretation of data. In this study; it is ensured by external audits conducted by the supervisors and one external expert on qualitative research. The confirmability is ensured when the research process is neutral and not influenced by the assumptions or biases of the researchers (Guba, 1981). In this study, this was ensured by audit trail.

### Research team and reflexivity

The principal investigator is a male physician who conducted all the interviews. At the time of the study, he was working with the Protestant University of Congo as a senior lecturer and had experience conducting qualitative inquiries. For this study, he had no previous relationship with the participants. The information about the researcher was shared with the participants for research purposes only. The principal investigator was assisted by two supervisors in conceptualizing this research, analysing the data and reporting the findings.

#### Ethical considerations

The study was approved by the Ethics Committee of the Protestant University of Congo (reference number: CEUPC 0067; date: 5 February 2021) and Human Research Ethics Committee (Medical) of the University of the Witwatersrand (reference number: M210308; date: 26 August 2021) and conducted according to the ethical guidelines of the Declaration of Helsinki. Permission was obtained from the Kinshasa Primary HealthCare Network to conduct the study. Before the interviews, the principal investigator provided study information summarized on a leaflet to potential study participants and obtained written informed consent. A copy of the leaflet and the signed consent form (to participate in the study and for audio recording) was given to the participant. Data collection was done with strict adherence to local COVID-19 regulations. The participants were not paid for their participation in the study. Nevertheless, the participants were reimbursed for the transport to come to the healthcare facilities. An informed consent for the publication of the study results was obtained from each participant.

They received an equivalent of 5$ US for the transport to the health facility.

An informed consent for publication of the study results was obtained from each participant.

## RESULTS

### General characteristics of included participants

Twenty-three participants were interviewed. The majority of the participants were female (60.87%) and of the 40–64 years age group (56.84%). About half of the participants had good glycaemic control (52.17%). The median duration of diabetes was 5 (IQR: 1.5–10) years. The general characteristics of the included participants are summarized in the Supplementary File 2.

### Themes identified for glycaemic control

The analysis of the data gathered provided a group of five themes as perspectives for glycaemic control for persons living with type 2 diabetes in Kinshasa. These themes are financial constraints, limited social and relational support, difficulties with lifestyle changes, beliefs about diabetes and the ability to adapt to what is required for caring for the illness. Each theme comprises sub-themes, which in turn contain initial codes (Table 1).

**Table 1:** Themes, sub-themes and codes identified as perspectives for glycaemic control in Kinshasa, Democratic Republic of the Congo

Theme	Sub-themes	Codes
Financial constraints	Unreliable financing	Scarcity of revenues
		Use of out-of-pockets payments
		Lack of health insurance
		Absence of support from the Government
		Irregular financial aid from family members
	Non respect of scheduled visits	Lack of money for visit fees
		Unable to afford indirect costs: transport….
	Insufficient number of tests for blood glucose	High cost needed for repeating the tests
		Fear to run out of strips
	Irregular meals	High cost of suitable meals for diabetes
		Self-adaptation of medications to avoid side effects
	Lack of money for medications	Stock run out of medications
		High cost of newer products
Limited social and relational support	Fear of the reaction from others	Sharing information about diabetes would result on altered relation with others
		Living differently is seldom accepted by others
	Inaccessibility of doctors between visits	Poor doctor–patient communication
		Limited number of trained personnel for treating diabetes
Difficulties with lifestyle changes	Difficulty due to physical and health condition or work schedule	Ageing and/or presence of Comorbidities or ageing limiting physical condition
		Workload letting few time or space for physical exercise
	Time needed to adopt the changes	Changes are difficult and need time
		Diabetes requires changes in many aspects of the life
	Confusing information on what to eat	Patients receiving sometimes contradictory advices from health providers
		Population beliefs on diet for diabetes
Beliefs and practices about diabetes	Diabetes has a mystic origin	Diabetes result from a bad sort
		Seeking traditional healers to cure diabetes
	Need for signs of increased blood glucose to take drugs	Presence of signs prompt to take medicines
		Absence of signs lead sometimes to neglect of treatment
Ability to adapt for what is required for caring the illness	Acquisition of skills	Persons with diabetes able to inject themselves
		Ability to use devices to follow-up glycaemia
		Persons with diabetes resolve difficulties related to the treatment of their illness
	Acquisition of knowledge about the disease	Ability to identify body alarms
		Knowing what the disease requires
	Encouragements from carers and relatives	Appreciated support from health workers
		Positive support from family members

### Theme 1: Financial constraints

Diabetes is a chronic disease requiring that at all times the person with the disease follows the treatment, takes the appropriate diet, regularly monitors his blood glucose and engages in follow-up scheduled visits. Financial constraints impact the capacity of the persons with diabetes to adequately follow these requirements.

A large proportion of the persons living with diabetes are not covered by health insurance and use out-of-pocket payments. They could only rely on their resources, which are often very limited. Most receive help from their family members, but this is sometimes irregular and unreliable. They regretted that they were not receiving the same support as patients suffering from HIV/AIDS or tuberculosis.

Sometimes, my family members help me with money when they have it. (P15, Male, 35 years)

Only one of my sons helps me financially. He doesn’t work and struggles to find money. The other family members don’t look after me. (P2, Female, 60 years)

Coming to scheduled visits also have indirect costs such as transport and the time they have to spend in the health centre. It is not unusual them had to wait for hours before seeing the doctor.

Sometimes, I do not have money for the transport as I’m living in Maluku and must come here for appointments. (P21, Male, 41 years)

The visits take too long. We spend about 5 hours for a visit. There are many patients coming here. (P2, Female, 60 years)

Many participants reported that they would take their medicines as prescribed provided the medicines are available. Indeed, many participants reported that they encountered difficulties in adhering to their treatment as they must first find money to buy drugs. Some participants are not regularly taking their treatments or personally change their regimen to allow their medicine stock to last longer.

Once I’m prescribed medicines by the doctor, I try to get the money as quickly as possible to buy these. Usually after three days, I can buy the medications. (P14, Female, 32 years)

Due to economic reasons, sometimes I don’t have to take the medicine exactly as prescribed to avoid a lot of expenses. (P10, Female, 40 years)

The financial constraints also limit their ability to perform blood glucose monitoring as they do not have money or the strips for those who have the glucometers.

The test for glycaemia is not free and is done for a fee. I only do it when I can pay. Otherwise, I won’t do it. (P2, Female, 60 years)

Testing regularly costs very much. In the beginning, I used to test my glycaemia every day. This was very difficult to follow. The medical team advised me to reduce to one test per week. (P15, Male, 35 years)

The persons with diabetes receiving insulin need to have regular meals to accompany the taking of their drugs. Many participants reported that they only take the medicines when they are sure that they will have something to eat to avoid side effects of the medications.

I would take the treatment only when I find something to eat. (P20, Male, 46 years)

Yes, sometimes if I don’t have enough food, I reduce the dose a little and then I want to go back to my usual routine. (P21, Male, 41 years)

If food is available, then I take the medicine. If not, I don’t take the medicine until the next day. When I have nothing to eat, I don’t take the medicine. (P2, Female, 60 years)

### Theme 2: Limited social and relational support

Participants expressed that diabetes brings emotional and financial upsets in persons with diabetes, for which external support is essential. The family members were the main people that provided some emotional support to participants. For persons advanced in age, a number were supported financially by their relatives to meet the cost of diabetes care. The family also contributed concerning diet. Some participants pointed out the roles of their relatives in reminding them to take their medicines. Other family members even helped in administering the medicines to the patients.

My father wants me to follow the treatment correctly. He advises me a lot. And sometimes he brings me the medicine when I have to take it. He always tells me that with the treatment, I will be better. (P19, Male, 41 years)

My third son got married to a girl who did medical science, she is the one who helps me with injections every days. Every morning and every evening before going to sleep. (P11, Male, 80 years)

If the closest family members were supportive of persons with diabetes, the participants reported that the people around them no longer thought of them as they used to once they knew they had diabetes. It is important to note that the general population does not know much about diabetes due to a lack of awareness or education on the subject. One participant even said that they were seen as condemned. As a result, they did not open up to anyone about their illness and only told those who were very close to them. One female participant expressed that while looking for a marriage, it would be difficult for her to be accepted if the partner knew that she was suffering from diabetes. Participants reported that, in some situations, the need to follow the requirements of diabetes care put them in an uncomfortable position.

People feel sorry for you as if it is the end of your life because diabetes has no cure. (P22, Male, 37 years)

I feel embarrassed when I go to a party, and I only have water, and people wonder why I just have water and no other drinks. (P9, Female, 51 years)

Yes, I’m thinking that diabetes is a real barrier to my wellness. I’m thinking that if I find a man who wants to marry me if I tell him that I have diabetes, he will leave me. Perhaps I will not tell the truth. But this will be difficult with the treatment that I have to follow. (P12, Female, 40 years)

In our study settings, the persons with diabetes are received once monthly; during these visits, they receive an education on a subject relating to their illness, have their blood glucose tested and are received by the doctor. It is important to note that during the monthly interviews, the waiting list is usually very long. Usually, there is one doctor to visit all the patients registered in the health centre and scheduled for the visit. The doctor is faced with the problem of not having enough time to devote to each person. The encounter covers rarely personal matters of living with the illness.

Moreover, apart from the monthly visit to the health centre, many participants expressed that they could not contact the doctor or nurse before the next meeting. When needed they could not adapt their regimens. Not all the nurses are trained in diabetes and able to make required adjustments to the treatments. They continue to follow the prescribed doses until the next scheduled appointment.

I cannot change (my treatment) until I come back here, I’m treated with insulin. (P14, Female, 32 years)

### Theme 3: Difficulties with lifestyle changes

Many barriers to regular physical activity were reported among participants. One participant reported that relating to her age, it would be difficult to engage in exercise. Some participants reported constraints of time as they must be at work. Their work is time-consuming and does not allow for exercise. Many finish work late and after that have to face lengthy traffic jams to arrive at home. Participants find it difficult to follow the diet. At the beginning of their disease, the participants must adapt to the requirements; after time many succeeded in adopting new meals.

It is very difficult for me because of my age. When the opportunity arises, I take a short walk in the corridors of the house. When I get tired, I give up. (P5, Female, 64 years)

I don’t know how to follow these recommendations properly because of my work rhythm. In the service, my schedule alternates between days on call and days off. I don’t know how to combine these shifts with my diet and exercise. (P4, Female, 55 years)

One participant stated that there was no accurate information on what the patients must eat. Sometimes, the advice was confusing—Some ailments have been prescribed by certain health providers while others were prohibiting them.

The advice we have from health providers is somewhat confusing. Sometimes, there is no precision on what we must eat. (P13, Male, 67 years)

### Theme 4: Beliefs and practices about diabetes

Some participants perceived that their diabetes could be due to a ‘spell’ or had another explanation than that offered by medical science, resulting in these participants experimenting with alternative medicines. However, some of these participants returned to their clinic prescriptions when they noticed that these alternative treatments did not work for them:

My family members think that diabetes in our family is a mysterious disease. There must be someone behind it. (P16, Male, 40 years)

They took me to a healer. I was mixing traditional treatment with pharmaceuticals. At one point, I even gave up the pharmaceuticals. But when the traditional treatment didn’t work, I came back to continue the treatment. (P17, Female, 35 years)

On the other hand, several participants recognized that when they feel well, they tend to abandon their treatment or to have a period of slackening:

I respect the treatment because I know that my health depends on it, despite some slackening. (P22, Male, 37 years)

I take my treatment regularly. But sometimes I neglect the treatment when I feel well. (P23, Female, 39 years)

### Theme 5: Ability to adapt to what is required for caring for the illness

Participants expressed their ability to effect requires change to attain good glycaemic control. This ability is conveyed by the acquisition of the technical skills to administer drugs daily and/or to monitor their glycaemia. And as time passes, it is easier to resolve issues emerging with the care. The ability to perform the change is also supported by the ability of the patient to know when s/he is experiencing signs of increased blood glucose and needs to take appropriate actions.

I care about taking the treatment for this disease. I have gained some experience in resolving the issues. (P15, Male, 55 years)

I have no problem using the glucometer. (P18, Female, 22 years)

When the glycaemia is high, you will know it by your body. (P12, Female, 40 years)

The participants reported that in the process of adapting to what their illness requires, they are helped by the positive messages conveyed by their surroundings and the healthcare providers.

The health providers said that diabetes will not end, but they make us understand that diabetes is a disease like the others and that it requires following the recommendations for treatment, diet and exercise. (P9, Female, 51 years)

## DISCUSSION

This study was designed to describe perspectives from persons living with type 2 diabetes for glycaemic control in Kinshasa, DRC. Five themes were identified: financial constraints, limited social and relational support, difficulties with lifestyle changes, beliefs and practices about diabetes and the ability to adapt to what is required for caring for the illness. Participants expressed they were unable to move towards better glycaemic control due to financial constraints, limited support and difficulty in changing their lifestyle. Their understanding of diabetes also constituted a barrier.

Diabetes is a chronic disease requiring lifelong and multidimensional care. Lack of resources was undermining many aspects of care for our participants and making them unable to access care and ensure self-care. An adequate system of funding is necessary to ensure that persons with diabetes benefit from their care, this is seldom possible in a setting marked by poverty (Adeniyi *et al.*, 2016; Mayer *et al.*, 2016; Okoronkwo *et al.*, 2016; Adisa *et al.*, 2017) necessitating an expanded social-support system for better health outcomes. The introduction of universal health coverage in Kinshasa will be of great benefit in ensuring equity of access to diabetes care (Jackson *et al.*, 2016; Stephani *et al.*, 2018). Many persons with diabetes currently rely on out-of-pockets payments that put them in a vicious circle where lack of money restricts care and leads to complications that will then require more money. Out-of-pocket payments are leading to poverty and efforts must be made to progressively suppress these (Tsega *et al.*, 2021). At the societal level and long term, it implies progressively empowering the people by a fight against poverty and engaging themselves with health mutuals. It is also important to act to reduce indirect costs such as for the transport to health structures with adequate diabetes care coverage. A study in South Africa showed that transport costs were contributing to over 50% of total healthcare costs (Mutyambizi *et al.*, 2019).

Participants expressed that family members were their sole source of social and treatment support. Social support is linked with better health outcomes including glycaemic control (Strom and Egede, 2012; Osuji *et al.*, 2018). The participants expressed concerns that reactions the others in their surroundings could manifest if they know about their illness or if they do not conduct themselves as the others. This behaviour could reflect the participants’ concern about maintaining a good image in the eyes of others and their fear of being stigmatized. Body image-related distress can lead to suboptimal glycaemic control (Shaban, 2010). The fact that the origin of diabetes is not well known in the population leaves room for many interpretations. As a result, persons with diabetes could not receive enough support. There is a need to educate the general public about diabetes, especially the people who live with patients so that they can better support people with the disease. Beyond the monthly visit, patients were rarely able to access doctors when they needed to discuss their treatment in between appointments. The limited doctor–patient communication may aggravate the effects of clinical inertia which is a recognized reason for failure to achieve glycaemic targets (Andreozzi *et al.*, 2020). Improving diabetes care also requires enhancing the skills of healthcare providers to enable them to develop a better approach to the management of chronic patients (Nang *et al.*, 2019). In our context where there is a chronic shortage of doctors, it may be unrealistic to expect doctors to be always available for all categories of patients with diabetes. Rather, nurse clinicians who are in constant contact with patients should be capacitated skills-wise to solve most of the common problems these patients present with.

Type 2 diabetes is a lifestyle disease and lifestyle modifications are the cornerstone of its management (Bekele *et al.*, 2020). Studies have shown that lifestyle interventions are more effective than standard care mainly when there is an association with weight loss (García-Molina *et al.*, 2020). A systematic review of lifestyle interventions on patients with type 2 diabetes suggested focusing on diet and physical activity as these had a significant effect on blood glucose and weight loss (Zhang *et al.*, 2023). In our study settings, it is particularly important to consider tailored interventions respective to the people’s medical conditions and economic situations. Moreover, it is crucial to advocate for the integration of workplace physical activity activities (To *et al.*, 2013).

The participants also expressed that the resurgence of the symptoms of hyperglycaemia prompted them to take their treatment. As stated earlier, there is a trend for some patients to abandon or neglect the treatment when they feel better. The resurgence of symptoms of hyperglycaemia needs to be considered as ‘teachable moments’ (Xiang, 2016) as they offer opportunities for strengthening health education such that patients understand the benefit(s) of maintaining good glycaemic control and that there is no benefit to wait for symptoms before being adherent to their treatments.

The beliefs of the participants—that diabetes is caused by human being’s influence, particularly witchcraft—represent a real threat to their lives, there is a need to provide sufficient and reliable information to help them develop a good understanding of diabetes disease. These beliefs could push persons with diabetes to orient themselves to ineffective alternative treatments, more so, that some of these are false promises of cure for diabetes. It is therefore crucial that healthcare providers assess their clients’ stage of change and motivate them to adopt the necessary lifestyle behavioural changes required for optimal diabetes care.

The participants expressed their ability to perform the required changes for good glycaemic control. Persons with diabetes are demanded to continuously resolve the issues related to living with diabetes and adopt the best solutions for their illness and its treatment. Care for people with diabetes must also seek to highlight the difficulties of adaptation to help patients resolve them (Kalra *et al.*, 2018). Studies showed that interventions for patient empowerment may improve glycaemia and blood pressure in persons with diabetes (Hood *et al.*, 2015; Mogueo *et al.*, 2020). In our study settings, this aspect of care has not received the required attention and needs to be considered when developing strategies for improving diabetes care in Kinshasa.

This study was based on a phenomenology approach which is premised on participants sharing their perspectives on glycaemic control (Rodriguez and Smith, 2018). Glycaemic control is a multifactorial issue (Fekadu *et al.*, 2019). Factors that have been reported in the literature to influence glycaemic control are related to the persons with diabetes, their relatives, the health system and the community/society (Zimmermann *et al.*, 2018; Fekadu *et al.*, 2019; Fina Lubaki *et al.*, 2022; Sagastume *et al.*, 2022).

Supplementary File 3 presents an integrated explanatory model for glycaemic control among persons living with type 2 diabetes in Kinshasa, DRC using the social cognitive theory. This theory posits that particular behaviour (such as effecting becoming adherent to treatment behaviours for maintaining good glycaemic control) is a product of interactions between personal, cognitive and contextual/environmental factors (Thojampa and Sarnkhaowkhom, 2019; Sebastian *et al.*, 2021). Put together, the five themes identified in our study are displayed about the six modules of social cognitive theory (Thojampa and Sarnkhaowkhom, 2019). Behavioural capacity refers to the patient’s ability to change a specific behaviour through the acquisition of essential knowledge and practices (Joseph *et al.*, 2017; Thojampa and Sarnkhaowkhom, 2019). The participants in our study expressed that they have learned about diabetes through previous experiences of diabetes with their relatives or from self-experience. Also, some of them have acquired the technical skills required for diabetes treatment (Theme 5). These achievements improved their self-efficacy in being able to do what is required for good glycaemic control (Joseph *et al.*, 2017; Thojampa and Sarnkhaowkhom, 2019). Self-efficacy refers to a person living with diabetes confidence in achieving good glycaemic control. Our results noted that participants were encountering many financial constraints in the care of their illness thus resulting in poor self-efficacy to achieve good glycaemic control (Theme 1). Reinforcements refer to internal or external responses to a given behaviour with a positive or negative impact on the continuity of the behaviour (Thojampa and Sarnkhaowkhom, 2019). Our results showed that participants lived in an unfavourable social context with limited social support (Theme 2) and were exposed to poor glycaemic control by the way they understood their illness (Theme 3) and the difficulties they had with lifestyle changes (Theme 4). These elements have a negative impact on glycaemic control.

Our explanatory model is aligned with social cognitive model as it is recognized that for persons with diabetes, it is inconceivable to change a behaviour without considering the environment (Thojampa and Sarnkhaowkhom, 2019). Among the modules of social cognitive theory, self-efficacy is a significant predictor of improved self-care among persons with diabetes who have poor health literacy; a feature of many persons with diabetes in our study setting (Reisi *et al.*, 2021). Social support is one of the main domains of social cognitive theory and, enhancing it in diabetes self-management could help persons with diabetes improve their glycaemic control, achieve self-management goals and facilitate coping with the disease (Thojampa and Sarnkhaowkhom, 2019).

Overall, this study is one of few studies in sub-Saharan Africa to investigate the perspectives of persons living with type 2 diabetes on glycaemic control and has clinical, public health and policy implications for improving glycaemic control in Kinshasa, DRC, and similar settings. The qualitative design has potential interviewer, selection and translation biases. However, the reflexivity of the principal researcher during interviews and analysis, the prior training in qualitative design and the oversight of an experienced qualitative researcher, all ensured the adoption of a neutral and non-judgmental posture and limited the potential for biases. Furthermore, it is rightly inherent in qualitative studies to sample information-rich participants. The participants’ statements could have been altered during translation from Lingala or French to English; nevertheless, the translation performed by the language expert was verified by the principal investigator by referring to the audio recordings. The findings of the study were submitted for peer review and member checking to enhance their credibility. A thick description of data and the context from which they emerged ensured the transferability, and external audits conducted by the supervisors, who are senior researchers, and one external expert on qualitative research ensured dependability.

In the future, engaging qualitative methods to understand the socio-cultural context underlying the persons with diabetes knowledge, attitudes, beliefs, perceptions and behaviours around diabetes will be of great help to improve the care of persons with diabetes.

## CONCLUSION

Our results showed that lack of adequate funding is a major determinant of glycaemic control. It is crucial to integrate a consistent and reliable funding system for the care of people living with diabetes. Persons with diabetes must be empowered to successfully adapt to the requirements of diabetes care. In this process, support for people living with type 2 diabetes is also essential and should involve their families as well as healthcare providers.

## Supplementary Material

daad128_suppl_Supplementary_Files_1

daad128_suppl_Supplementary_Files_2

daad128_suppl_Supplementary_Files_3
