# Supplementary material for: Perspectives for glycaemic control in type 2 diabetes in Kinshasa, Democratic Republic of the Congo
Source: Health Promot Int. 2023 Oct 10;38(5):daad128. doi: 10.1093/heapro/daad128 (PMC10563016; doi:10.1093/heapro/daad128)
Supplement: daad128_suppl_Supplementary_Files_1 [file daad128_suppl_supplementary_files_1.pdf]

SUPPLEMENTARY FILE 1. INTERVIEW GUIDE FOR IN-DEPTH INTERVIEW

Topic I: Self-management of diabetes

What was your reaction when you were told you have diabetes?
What were you told about diabetes mellitus?
What were you told about self-management?
What are your feelings about self-management of diabetes?
Can you tell us what you typically eat?
What kind of effect you think diet has on your diabetes?
How do you monitor your glucose?
How do changes in your treatment are made?
Tell us about your exercise plan for a typical week
Why is the diabetes treatment important for you?
What are your therapeutic goals?
How do you describe your commitment to the treatment?
What changes did you have to make to your daily life?
What is your motivation to follow the treatment?
What are the complications that you have been told during education? How to avoid theses?

Topic II: Support from family/friends/others

Who are the most supportive persons since you have been told you have diabetes?
Can you describe the kind of support they offer?
Can you tell us how following diabetes treatment place you in uncomfortable position with your entourage?

Topic III: Community

What are the thoughts of the members of your community about diabetes?
What do you think are the consequences of these thoughts on your diabetes care?
Describe where you access your treatment for diabetes mellitus
Can you tell us how following the treatment puts you in difficulty in work? Waiting lists, number of appointments, distance from home to health center…

Topic IV: Healthcare system
4.1 Accessing diabetes treatment
Do you have any challenge regarding attending the facility for your treatment?

Describe the health facility you are attending for your diabetes mellitus

Tell us about your relation with the healthcare providers

4.2 Health insurance

Tell us how diabetes treatment interferes with your usual expenses

What are the competing priorities do you have to deal in following your treatment?

4.3 Communication with the health providers/health facility

Tell us more about your relation with the healthcare providers.

What are the challenges that make it difficult for you to respond to scheduled appointments or to follow treatment?

Describe a typical encounter with the healthcare provider during scheduled appointment

How the health facility where you are receiving diabetes care is organized?

Topic V:

Food security

Tell us about your experience on accessing food before and after being diagnosed with diabetes.

Topic VI: Closing the interview

Additional comments

Acknowledgements
