# Supplementary material for: Perspectives for glycaemic control in type 2 diabetes in Kinshasa, Democratic Republic of the Congo
Source: Health Promot Int. 2023 Oct 10;38(5):daad128. doi: 10.1093/heapro/daad128 (PMC10563016; doi:10.1093/heapro/daad128)
Supplement: daad128_suppl_Supplementary_Files_2 [file daad128_suppl_supplementary_files_2.pdf]

Supplementary file 2. General characteristics of included participants, N=23

	Categories	n (%)
Age (years)	32-39	7 (30.4)
	40-64	13 (56.5)
	≥65	3 (13.0)
Sex	Female	14 (60.87)
	Male	9 (39.13)
Control of glycaemia	Poor	11 (47.83)
	Good	12 (52.17)
Duration of diabetes	Median (IQR), years	5 (1.5-10)
Treatment regimen	Insulin	5 (21.74)
	Insulin plus Oral hypoglycaemic agents	7 (30.43)
	Oral hypoglycaemic agents	11 (47.83)
