# Supplementary figures and images for: Perspectives for glycaemic control in type 2 diabetes in Kinshasa, Democratic Republic of the Congo
Source: Health Promot Int. 2023 Oct 10;38(5):daad128. doi: 10.1093/heapro/daad128 (PMC10563016; doi:10.1093/heapro/daad128)

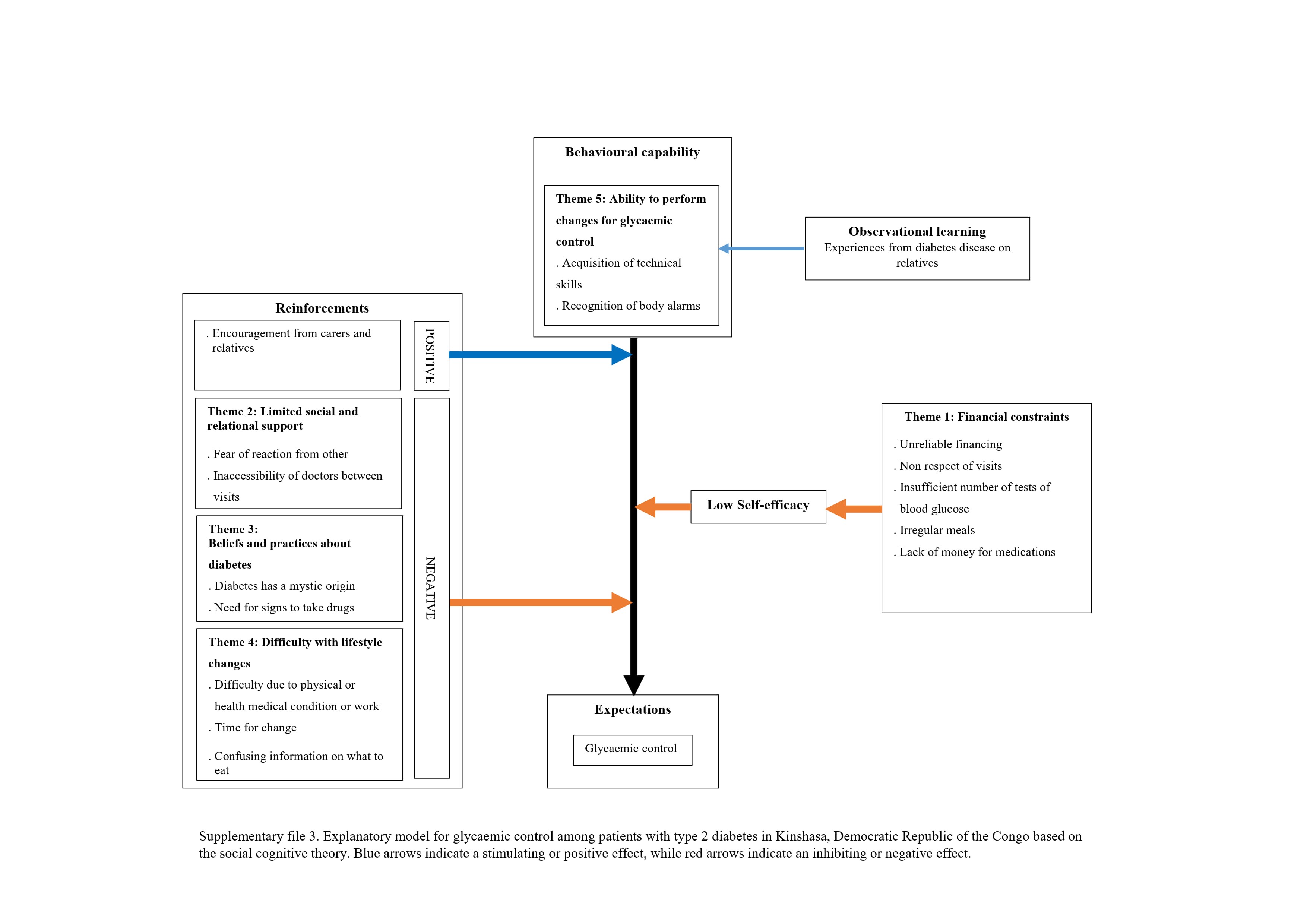

Supplement: daad128_suppl_Supplementary_Files_3 [file daad128_suppl_supplementary_files_3.jpeg]
